# Drip irrigation affects soil bacteria primarily through available nitrogen and soil fungi mainly via available nutrients

**DOI:** 10.3389/fmicb.2024.1453054

**Published:** 2024-11-27

**Authors:** Xiaojuan Wang, Yu Zhang, Tianle Wang, Lei Wang, Enke Liu

**Affiliations:** ^1^Shanxi Institute of Organic Dryland Farming, Shanxi Agricultural University, Taiyuan, Shanxi, China; ^2^College of Agriculture, Shanxi Agricultural University, Taigu, Shanxi, China; ^3^State Key Laboratory of Integrative Sustainable Dryland Agriculture, Shanxi Agricultural University, Taiyuan, Shanxi, China; ^4^Key Laboratory of Sustainable Dryland Agriculture (Co-construction by the Ministry of Agriculture and Rural Affairs and Shanxi Province), Shanxi Agricultural University, Taiyuan, Shanxi, China; ^5^Shanxi Province Key Laboratory of Sustainable Dryland Agriculture, Shanxi Agricultural University, Taiyuan, Shanxi, China

**Keywords:** maize, soil nutrients, soil microorganisms, drip irrigation, correlation and redundancy analysis

## Abstract

The issue of water scarcity is a global concern. Water-saving irrigation has long been a topic of interest among agricultural researchers. In this study, changes in soil microbial community structure and diversity under different periods of drip irrigation were analyzed using the Illumina HiSeq high-throughput sequencing platform and 16S rRNA gene sequence amplification. Six treatments were established based on varying drip irrigation amounts: maintaining the drip irrigation amount at 320 mm without any increase (CK), increasing by 72 mm during different growth stages: from the sowing stage to the jointing stage (J), from the jointing stage to the big trumpet stage (B), from the big trumpet stage to the tasseling stage (T), from the tasseling stage to the grain filling stage (G), and from the grain filling stage to the maturity stage (M). Compared to CK, the T treatment significantly increased the Chao index of soil bacteria by 2.95%. The main bacterial phyla included Proteobacteria, *Acidobacteria*, *blastomonas, Actinobacteria*, *Chloromycetes*, and *Bacteroidetes*, while *ascomycetes*, *basidiomycetes*, *chytridomycetes,* and *mortieromycetes* were the main fungal phyla across different periods of drip irrigation. *Zoopagales*, *Amtridomyces*, and *Trichomyces* were absent in the G, T, and M treatments, respectively. The content of soil-available potassium in the T treatment was higher than that in other treatments, whereas the content of soil-available nutrients in the B treatment was the lowest. Overall, the T treatment had the highest content of available nutrients. Redundancy analysis showed that available nitrogen was the main soil chemical property affecting soil bacterial community structure, while soil-available nutrients were the main soil chemical property affecting the fungal community structure. Thus, the T treatment was effective in enhancing soil microbial community structure and increasing soil-available nutrients.

## 1 Introduction

Water scarcity restricts sustainable economic and social development. In China, agricultural irrigation accounts for 63% of total water usage, making it the primary source of water consumption (Chen et al., 2018). The total water resources in Shanxi Province amount to 12.38 billion m^3^, influenced by complex terrain and meteorological factors. Drought has become a significant factor affecting crop growth, and the use of water-saving irrigation technology can yield both economic and ecological benefits, promoting sustainable agricultural development (Du, 2020). Common water-saving irrigation methods include border, furrow, drip, infiltration, and micro-spray irrigation. Drip irrigation is widely used in cash crop planting due to its water-saving advantages, high efficiency, precision, and low cost (Su, 2021). Compared to drip irrigation combined with mulching film, drip irrigation without mulching film reduces input costs, avoids subsequent environmental pollution, and is suitable for most areas (Yang et al., 2020). In 2018, drip-irrigated food crops accounted for 78% of the irrigated areas in China (Yin, 2018).

The grain planting area in China initially declined but later increased. The cultivated area of maize in Shanxi Province has consistently held the leading position within the province in terms of the cultivated area of grain crops (Yuan et al., 2020). The rate of increase in maize yield surpasses that of millet and wheat (Zheng and Cheng, 2021). To better meet the growing demand for maize, it is important to use drip irrigation technology to develop maize cultivation.

Different drip irrigation modes have varying impacts on crops and the soil environment. As the main influencing factors, the amount and frequency of drip irrigation can influence water use efficiency (Li et al., 2021) and nutrient absorption rate (Zhang et al., 2021). Furthermore, they have the potential to alter the spatial distribution of crop roots (Tian et al., 2021), sustain root vitality in the later growth stages (Xue et al., 2022), and modulate the abundance and composition of soil microorganisms in a beneficial manner, thereby enhancing both crop quality and yield (Tang et al., 2020). Soil, crops, and microorganisms interact with one another in important ways. As the most active component of the soil ecosystem, soil microorganisms are also the most sensitive biological indicators of soil quality. Their small size, large populations, and diverse species significantly impact material decomposition, soil fertility, and crop productivity. Moreover, they actively participate in material cycling and energy flow (Song et al., 2013). Soil nutrient status affects soil microbial diversity and community composition (Lauber et al., 2008). A study conducted by Dai et al. (2020) revealed that microorganisms play a pivotal role in soil phosphorus cycling, with key functions including the activation, transport, and regulation of phosphorus. An increase in soil salinity has been demonstrated to significantly reduce the relative abundance of soil bacteria, fungi, and *Actinomycetes* (Niu et al., 2017).

Furthermore, research indicates that the use of drip irrigation can reduce the risk of secondary salinization due to deep infiltration (Zhai et al., 2023). Dai and Miao (2019) concluded that nitrifying and denitrifying microbial communities in the Yellow River Delta were significantly influenced by soil water content and quick phosphorus content. Therefore, the effects of increasing drip irrigation at different growth stages of maize on crop yield, soil-available nutrients, soil bacteria, and fungi were analyzed. These effects are significant for improving the soil environment and achieving sustainable development. This research employed a high-throughput sequencing method to investigate the diversity of bacteria and fungi in the soil, track changes in bacterial species and their relative abundance at different classification levels, and assess the correlation between soil bacteria and surrounding environmental factors.

## 2 Materials and methods

### 2.1 Test site profile

The experiment was conducted at Dongyang Base of Shanxi Agricultural University in 2021. The test site is located in Dongyang Town, Yuci District, Jinzhong City, Shanxi Province (112°40 ′5 “E, 37°33′ 22′′ N), with an altitude of 802 m. The annual average precipitation was 410–490 mm. Rainfall is mostly concentrated in the summer. The region is a typical semi-arid area of the Loess Plateau, characterized by cold, dry winters and an annual average temperature of 9–10°C. The terrain in this area was flat. The soil is classified as loess brown soil. The groundwater is deeply buried and does not affect the experiment. In this region, natural rainfall is often supplemented with simple drip irrigation. The rainfall during the maize growth period during the experiment year was 322 mm.

### 2.2 Experimental design

The experiment utilized a randomized block design with single-factor control to vary irrigation amounts during different maize growth periods. A total of 6 treatments were set up (Table 1), and each treatment was repeated three times. These treatments included maintaining the drip irrigation amount at 320 mm without any increase (CK), increasing by 72 mm from the sowing stage to the jointing stage (J), from the jointing stage to the big trumpet stage (B), from the big trumpet stage to the tasseling stage (T), from the tasseling stage to the grain filling stage (G), and from the grain filling stage to the maturity stage (M). The plot area was 25 m^2^. An approximately 15 cm-thick cement layer, extending 3 m deep, was installed around each plot to prevent irrigation water seepage into neighboring plots. To avoid being affected by natural rainfall, the maize was planted in automatic rainsheds. The previous crop in the test field was maize, and its fertilization levels were administered in accordance with customary local practices. The drip irrigation belt was placed on the surface, 8–10 cm away from the maize root, and the drip irrigation frequency was 10 days/time. Before sowing, base fertilizer (N 150 kg/hm^2^, P_2_O_5_ 60 kg/hm^2^) was evenly applied to each plot, and the soil was tilled to a depth of 10 cm using a small rotary tiller. The planting amount was 60,000 plants /hm^2^. The sowing depth was 4–5 cm. Field preparation and fertilization were completed on 2nd May, sowing occurred on 13th May, and harvesting took place on 30th September. The maize variety tested (*Zea Mays* L.) was “dafeng 30.”

**Table 1:** Processing settings for the experiment.

Treatments	Drip irrigation volume (mm)					
	Sowing—Jointing stages	Jointing—Big trumpet stages	Big trumpet—Tasseling stages	Tasseling—Grain filling stages	Grain filling—Maturity stages	Whole growth period
CK	72	72	72	72	72	360
J	168	72	72	72	72	456
B	72	168	72	72	72	456
T	72	72	168	72	72	456
G	72	72	72	168	72	456
M	72	72	72	72	168	456

CK, J, B, T, G, and M represent no increase in drip irrigation amount, an increase in drip irrigation amount from the sowing stage to the jointing stage, from the jointing stage to the big trumpet stage, from the big trumpet stage to the tasseling stage, from the tasseling stage to the grain filling stage, and from the grain filling stage to the maturity stage of maize, respectively.

### 2.3 Soil sample collection for bacterial and soil chemical analysis

After crop harvest, the soil was sampled once, and the mixed soil was collected from each plot using a soil drill in the 0–20 cm soil layer according to the five-point sampling method. This process was conducted for six treatments, with three replicates in each group, resulting in 18 soil samples. Impurities such as stones and roots were removed from the soil samples. Half of the soil was sieved using a 2-mm diameter sieve. Then, the soil sample was put into a sealed bag and temporarily stored with dry ice. It was brought back and stored in the −80°C ultra-low temperature refrigerator to measure soil bacteria and fungi’s quantity, diversity, and community structure. The remaining soil was placed in a cool, ventilated area until completely dried. Then, the soil was ground, sieved with a diameter sieve of 1 mm, and stored at room temperature in a self-sealing bag. The contents of available nitrogen, phosphorus, and potassium were determined using the alkaline diffusion method (Shang et al., 2022), molybdenum-antimony resistance colorimetry (Zhang and Wang, 2022), and flame spectrophotometry, respectively (Zhang and Wang, 2022).

A quantity of 0.5 g of the sample was added to a centrifuge tube containing 500 μL of buffer SA, 100 μL of buffer SC, and 0.25 g of grinding beads. The soil samples were mixed and centrifuged using a TGrinder H24 tissue grinding homogenizer. The buffer was added to the supernatant on multiple occasions and subsequently centrifuged. Subsequently, 700 μL of deproteinizing solution and magnetic bead suspension were added, and the centrifuge tubes were placed on a magnetic rack to allow the magnetic beads to draw off the liquid. Subsequently, 50–100 μL of elution buffer TB was added, resulting in the retrieval of the DNA solution following the complete adsorption of the magnetic beads. Bacteria and fungi have been observed to encode ribosomal RNA based on conserved regions of nucleic acid sequences in the 16S and ITS regions, respectively. Subsequently, the sequences were subjected to high-throughput sequencing on the Illumina Novaseq platform. Firstly, Trimmomatic was used to filter the quality of the raw data, followed by the identification and removal of primer sequences by Cutadapt. USEARCH was used to control the quality of the raw sequences to obtain high-quality sequences. The high-quality sequences were clustered and then divided into OTUs.

### 2.4 Data processing and calculation methods

Excel 2003 software, high-throughput sequencing platform, Trimmomatic (version 0.33), Cutadapt software, and Usearch v10 were used to process the data. SPSS 18.0 software was used for statistical analysis, and QIIME2 software was used for Alpha index analysis. R language tools were utilized for drawing.

## 3 Results

### 3.1 Soil-available nutrients

Soil-available nitrogen content in the B treatment was significantly lower than that in the CK, J, and M treatments by 30.77, 33.40, and 35.84%, respectively (Table 2). The contents of available nitrogen with J and M treatments increased by 3.95 and 7.90%, respectively, compared to CK. No significant differences were observed in soil-available phosphorus content. Soil-available potassium content with T treatment was significantly higher than with CK, J, B, G, and M by 33.54, 17.17, 35.65, 27.98, and 31.50%. The content of soil-available nitrogen, phosphorus, and potassium with B treatment was the lowest, only 158.50 mg/kg. The content of comprehensively available nutrients with T treatment was more prominent.

**Table 2:** Effects of different drip irrigation treatments on soil-available nutrients.

Treatments	Available nitrogen (mg/kg)	Available phosphorus (mg/kg)	Available potassium (mg/kg)
CK	24.05a	26.00a	161.00b
J	25.00a	31.95a	183.50b
B	16.65b	23.90a	158.50b
T	20.35ab	29.55a	215.00a
G	20.35ab	26.15a	168.00b
M	25.95a	28.00a	163.50b

CK, J, B, T, G, and M represent no increase in drip irrigation amount, an increase in drip irrigation amount from the sowing stage to the jointing stage, from the jointing stage to the big trumpet stage, from the big trumpet stage to the tasseling stage, from the tasseling stage to the grain filling stage, and from the grain filling stage to the maturity stage of maize, respectively. The values adjacent to the mean values were standard deviations. In the same column, different small letters refer to differences among treatments at *p* < 0.05 level.

### 3.2 Soil sample sequencing results and sampling depth verification

Quality control was performed on the original sequencing sequences to obtain high-quality sequences. The high-quality sequences were classified into OTUs after clustering, and the data were evaluated by statistical parameters such as sequence number and sequence length at each stage. The sequencing results showed that each sample of bacteria produced an average of 79,703 raw reads and 77,832 effective reads, or at least 77,410 effective reads, with an average sequence length of 420 bp. The sample sequence was sufficient for data analysis. Each sample of fungi produced an average of 79,995 original sequences and 77,989 effective sequences. The main distribution range of sequence length was wide, ranging from 160 to 460 bp, and the average sequence length was 268 bp. The sample sequences were sufficient for data analysis. Sequences were clustered at 97% similarity to form operational taxonomic units (OTUs), each representing a classification unit identified by the analyst with a corresponding representative sequence. The number of OTUs can reflect the diversity of soil bacteria and fungi to a certain extent. There was no significant difference in the effective sequence readings of bacteria and fungi and the number of OTUs among all treatments, whereas the difference in bacterial OTUs was small, and the order from high to low was G > M > J > T > B > CK (Table 3). Fungal OTUs were ranked as T > G > M > B > J > CK from high to low, and the variation range of each treatment was 0.99–6.49% compared to CK. The OTU number of bacteria and fungi with CK was lower than that with other treatments, and increasing irrigation amounts during any maize growth period could raise the number of soil microorganism OTUs. The OTU number of fungi was significantly lower than that of bacteria, which was approximately 1/4 of that of bacteria. Increasing drip irrigation had a greater effect on the variation range of soil fungi.

**Table 3:** Effective reads and OTU numbers of soil microorganisms in the different drip irrigation treatments.

Treatments	Bacteria		Fungus	
	Effective reads	OTUs	Effective reads	OTU numbers
CK	77675.00	1621.50	78224.00	454.50
J	77794.00	1652.00	78182.50	459.00
B	77883.00	1639.50	76737.50	476.50
T	78045.50	1648.00	78317.50	484.00
G	77788.00	1656.00	78086.50	481.50
M	77804.00	1654.50	78386.00	479.00

CK, J, B, T, G, and M represent no increase in drip irrigation amount, an increase in drip irrigation amount from the sowing stage to the jointing stage, from the jointing stage to the big trumpet stage, from the big trumpet stage to the tasseling stage, from the tasseling stage to the grain filling stage, and from the grain filling stage to the maturity stage of maize, respectively.

**Figure 1:**
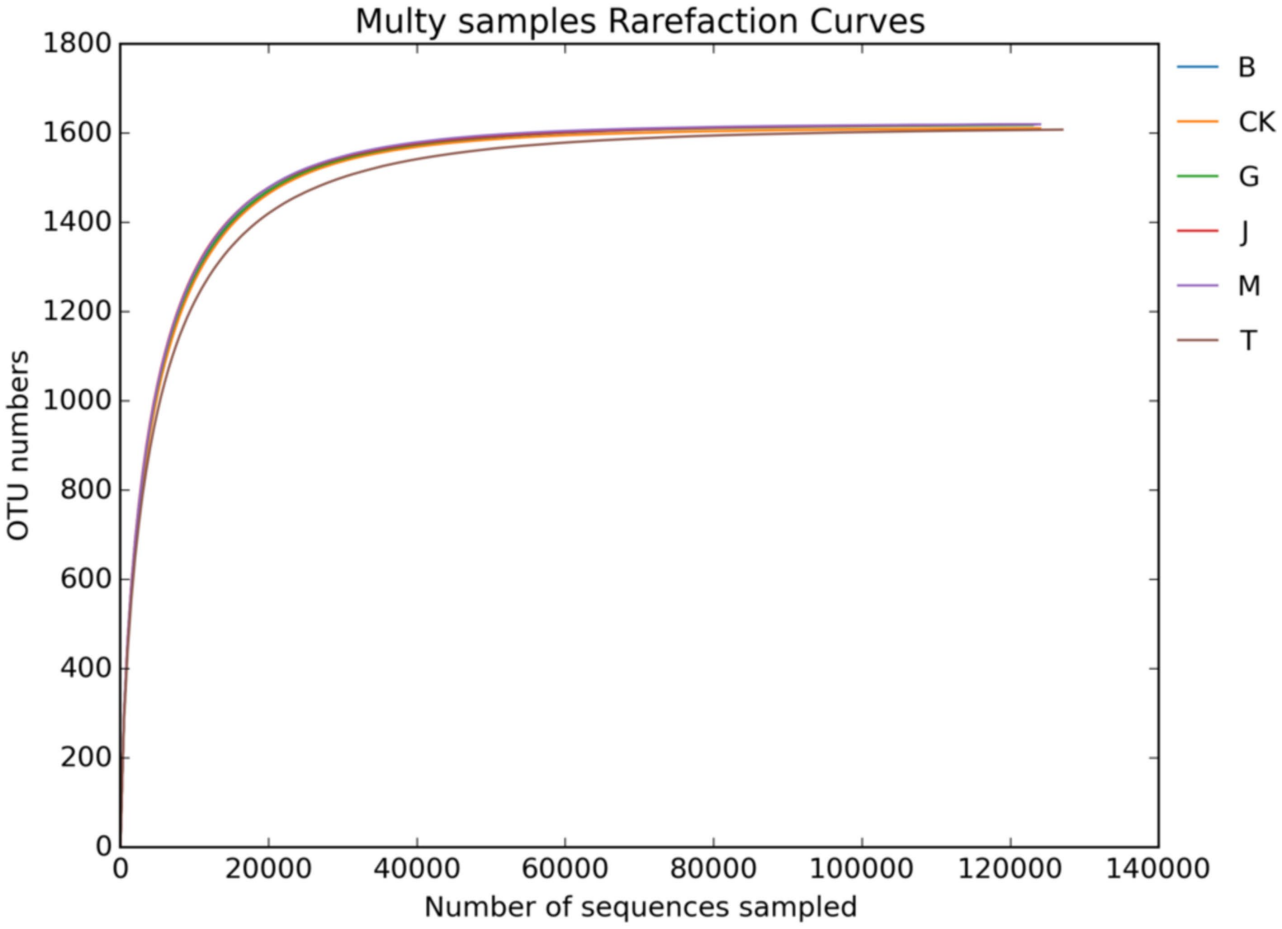
Soil bacterial dilution curve. CK, J, B, T, G, and M represent no increase in drip irrigation amount, an increase in drip irrigation amount from the sowing stage to the jointing stage, from the jointing stage to the big trumpet stage, from the big trumpet stage to the tasseling stage, from the tasseling stage to the grain filling stage, and from the grain filling stage to the maturity stage of maize, respectively.

**Figure 2:**
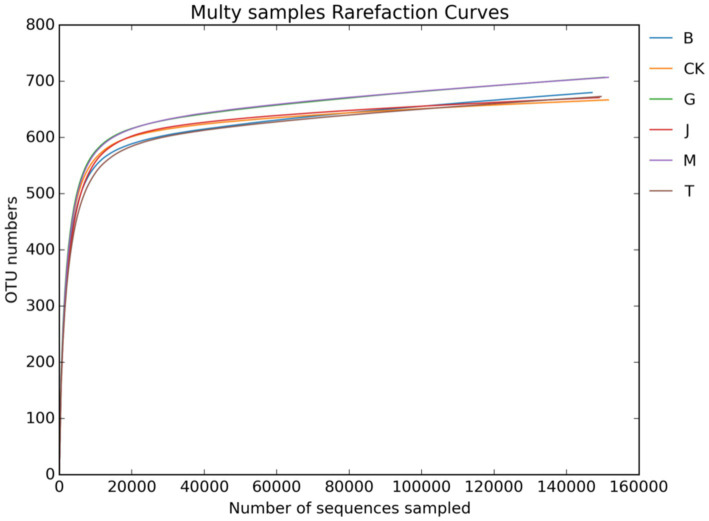
Soil fungal dilution curve. CK, J, B, T, G, and M represent no increase in drip irrigation amount, an increase in drip irrigation amount from the sowing stage to the jointing stage, from the jointing stage to the big trumpet stage, from the big trumpet stage to the tasseling stage, from the tasseling stage to the grain filling stage, and from the grain filling stage to the maturity stage of maize, respectively.

Figures 1, 2 show the dilution curves of bacteria and fungi for each treatment at 97% similarity. As the number of sequences increased, the number of OTU leveled off, indicating that the sampling had adequately covered all species in each treatment and accurately reflected the composition of the soil microbial community.

### 3.3 Soil microbial alpha diversity

The Alpha diversity index can reflect species richness and species diversity. Species richness represents the number of species, that is, the estimate of the number of OTUs contained in the treatment. The ACE, Chao, and Simpson index values for soil bacteria in the CK treatment were the lowest (Table 4). There was no significant difference in Shannon index value among all treatments. The Chao index value for the T treatment was significantly higher than that of CK. Moreover, the ACE value of the T treatment was the highest among all treatments, indicating that the number of soil bacteria under the T treatment was the highest. There was no significant difference in ACE, Chao, Simpson, and Shannon index values of soil fungi. The Chao index of T treatment was significantly higher than that of CK. Soil bacterial species richness was highest in the T treatment, while soil fungal species richness and diversity were highest in the G treatment.

**Table 4:** Alpha diversity index of soil microorganisms under different drip irrigation treatments.

Treatment	Bacterial alpha diversity index				Fungal alpha diversity index			
	Richness index		Diversity Index		Richness index		Diversity Index	
	ACE	Chao1	Simpson	Shannon	ACE	Chao1	Simpson	Shannon
CK	1636.82a	1639.59b	0.9962a	9.12a	569.12a	570.21a	0.9744a	6.76a
J	1666.53a	1677.42ab	0.9966a	9.23a	514.04a	518.44a	0.9504a	6.29a
B	1662.32a	1678.48ab	0.9967a	9.23a	688.01a	578.41a	0.9706a	6.64a
T	1674.34a	1688.03a	0.9962a	9.08a	663.40a	666.77a	0.9697a	6.58a
G	1676.13a	1685.82ab	0.9963a	9.17a	748.86a	667.41a	0.9775a	6.88a
M	1672.79a	1677.82ab	0.9963a	9.19a	709.86a	609.12a	0.9688a	6.62a

CK, J, B, T, G, and M represent no increase in drip irrigation amount, an increase in drip irrigation amount from the sowing stage to the jointing stage, from the jointing stage to the big trumpet stage, from the big trumpet stage to the tasseling stage, from the tasseling stage to the grain filling stage, and from the grain filling stage to the maturity stage of maize, respectively. In the same column, different small letters refer to differences among treatments at *p* < 0.05 level.

### 3.4 Soil bacterial community structure

**Figure 3:**
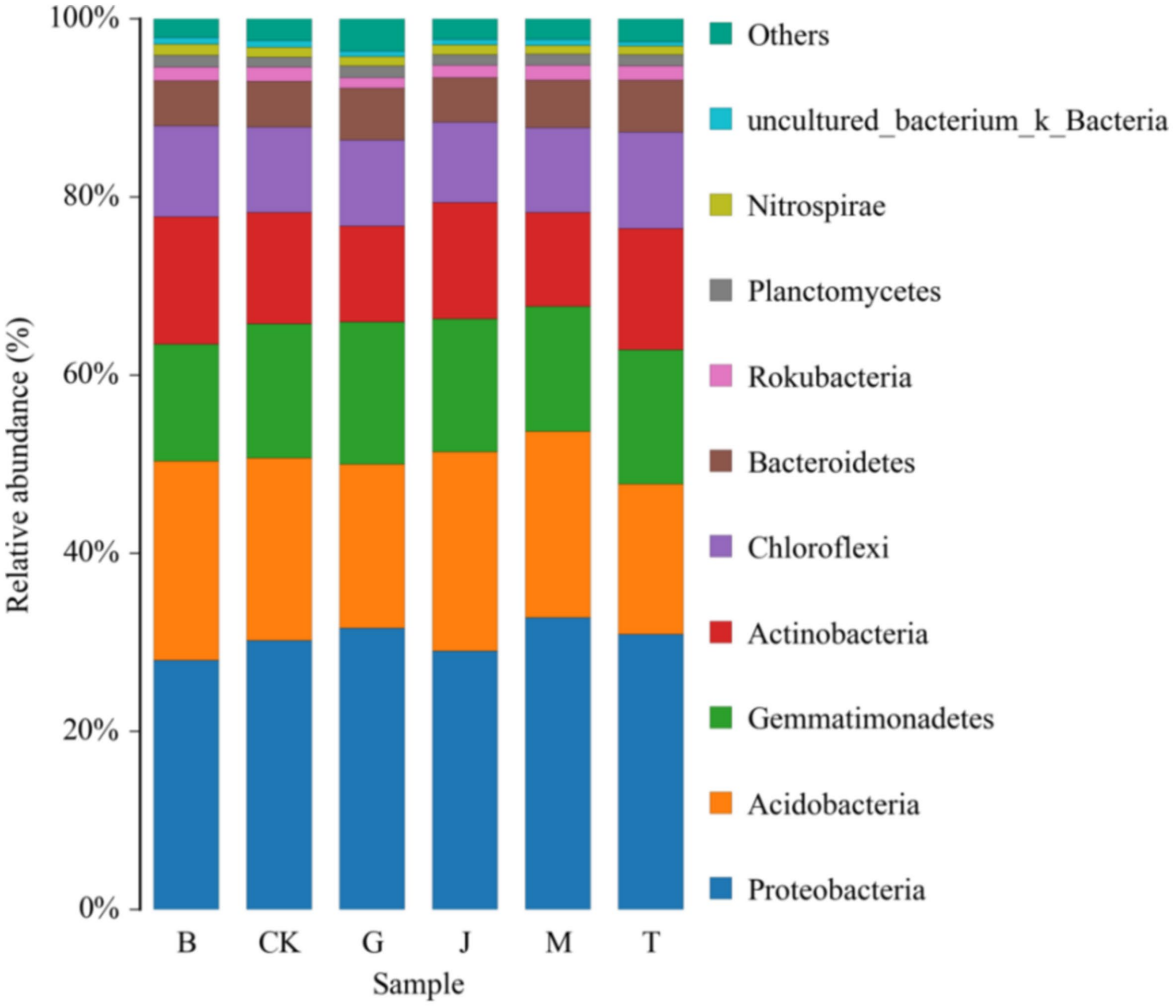
Community composition of bacteria at phylum level in the different treatment soils. CK, J, B, T, G, and M represent no increase in drip irrigation amount, an increase in drip irrigation amount from the sowing stage to the jointing stage, from the jointing stage to the big trumpet stage, from the big trumpet stage to the tasseling stage, from the tasseling stage to the grain filling stage, and from the grain filling stage to the maturity stage of maize, respectively.

*Proteobacteria*, *Acidobacteria*, *Gemmatimonadetes*, *Actinobacteria*, *Chloromyces,* and *Bacteroidetes* were the main bacterial groups in the soil of each treatment, and the sum of their relative abundance accounted for more than 92.00% of the total bacteria (Figure 3; Table 5). *Proteobacteria* exhibited an initial decrease followed by an increase as irrigation was applied later in the growth stages. The M treatment led to a significant 17.20% increase in Proteobacteria compared to the B treatment. *Gemmatimonadetes* exhibited an initial decrease, followed by an increase, and then a subsequent decrease as irrigation progressed. The G treatment significantly increased *Gemmatimonadetes* by 21.05% compared to B treatment. *Actinobacteria* increased first and then decreased as the irrigation time moved back. The M treatment significantly reduced Actinobacteria by 35.27% compared to the B treatment.

**Table 5:** The difference in the relative abundance of bacteria at the level of phylum classification.

Treatments	Relative abundance (%)						
	Proteobacteria	Gemmatimonadetes	Actinobacteria	Rokubacteria	Planctomycetes	Nitrospirae	uncultured bacterium
CK	30.12ab	15.26ab	12.43ab	1.55ab	1.12c	1.08ab	0.79a
J	28.98ab	15.09ab	13.08ab	1.40ab	1.18bc	1.07ab	0.64ab
B	27.96b	13.30b	14.23a	1.51ab	1.36a	1.24a	0.76a
T	30.85ab	15.32ab	13.53ab	1.62a	1.21abc	1.03ab	0.46b
G	31.61ab	16.10a	10.74ab	1.15b	1.31ab	1.07ab	0.57ab
M	32.77a	14.13ab	10.52b	1.64a	1.26abc	0.99b	0.70ab

CK, J, B, T, G, and M represent no increase in drip irrigation amount, an increase in drip irrigation amount from the sowing stage to the jointing stage, from the jointing stage to the big trumpet stage, from the big trumpet stage to the tasseling stage, from the tasseling stage to the grain filling stage, and from the grain filling stage to the maturity stage of maize, respectively. In the same column, different small letters refer to differences among treatments at *P* < 0.05 level.

The relative abundance ratio of T and M treatments in *Hexaceae* was significantly higher than that of G treatments, and only T and M treatments were higher than CK. *Nitro Spirillaria* and *Hexaceae* were closely related, and the Nitro Spirillaria with B treatment was significantly higher than that with M treatment.

*Planctomycetes* levels in the B treatment were significantly higher than those in the J treatment. B and G treatments significantly increased Planctomycetes compared to CK.

### 3.5 Effects of different drip irrigation treatments on soil fungal community structure

**Figure 4:**
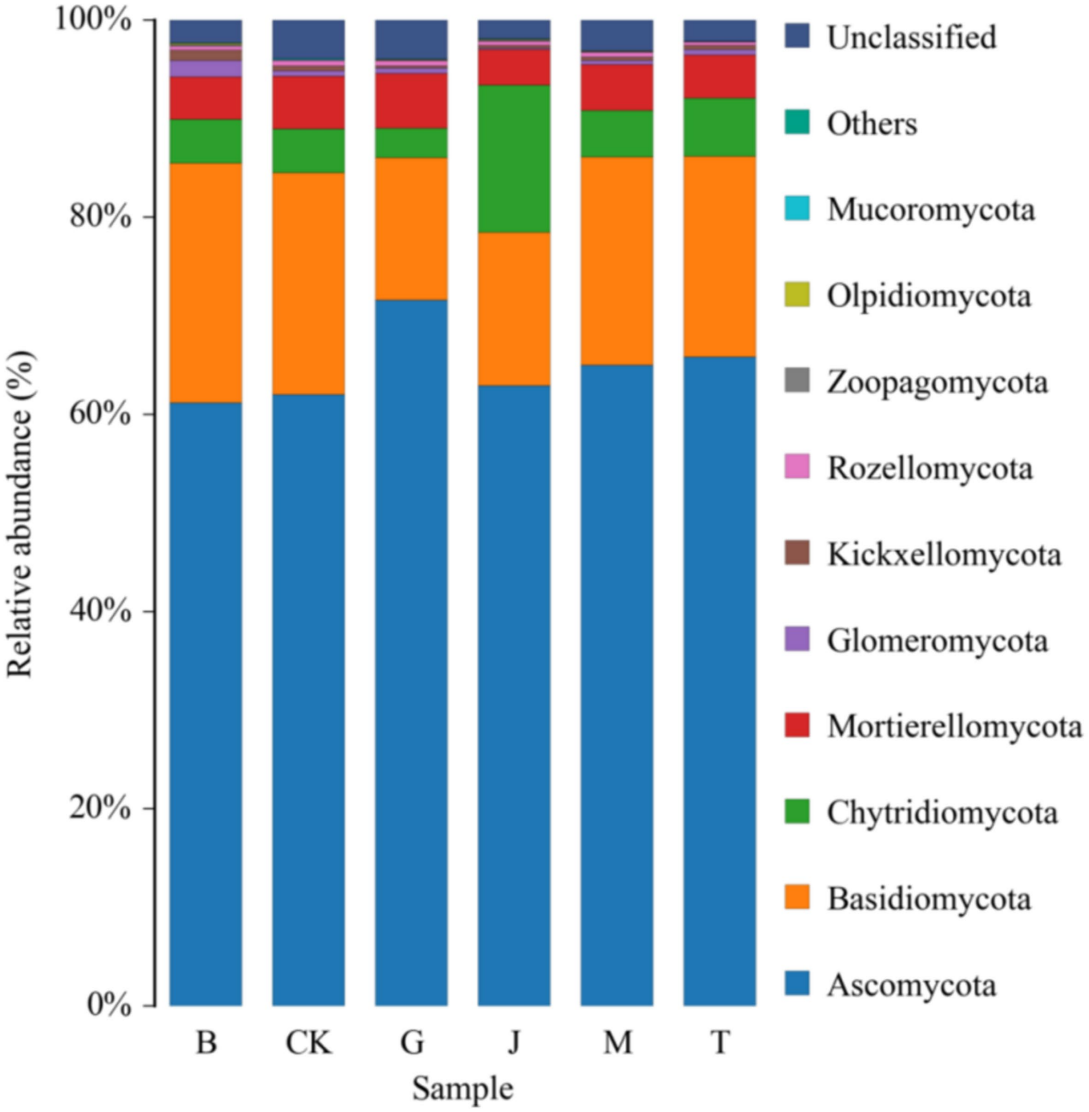
Community composition of fungus at phylum level in the different treatment soils. CK, J, B, T, G, and M represent no increase in drip irrigation amount, an increase in drip irrigation amount from the sowing stage to the jointing stage, from the jointing stage to the big trumpet stage, from the big trumpet stage to the tasseling stage, from the tasseling stage to the grain filling stage, and from the grain filling stage to the maturity stage of maize, respectively.

The relative abundance of *Sphaerospora* in the B treatment was significantly higher than in other treatments (Table 6). The order of relative abundance from highest to lowest was *Ascomycota, Basidiomycota, Chytridiomycota, Mortier ellomycota, Spheromycota, Pectomycota Kickxellomycota, Cryptomycota Rozellomycota,* trap-worm *Zoopagomycota, Olpidiomycota* and *Mucoromycota*, which accounted for more than 96.00% of the total number of fungi (Figure 4). *Zoopagales* was absent in the G treatment but was found in the other treatments. *Amtridomyces* were not found in the T treatment but were detected in the remaining treatments. *Trichomyces* was not found in the M treatment but was present in the remaining treatments.

**Table 6:** Relative abundance of fungus at the level of phylum classification.

Treatments	Relative abundance (%)									
	Ascomycota	Basidiomycota	Chytridiomycota	Mortierellomycota	Glomeromycota	Kickxellomycota	Rozellomycota	Zoopagomycota	Olpidiomycota	Mucoromycota
CK	62.06a	22.49a	4.38a	5.36a	0.56b	0.50a	0.55a	0.07a	0.02a	0.12a
J	62.95a	15.60a	14.85a	3.60a	0.19b	0.25a	0.40a	0.19a	0.03a	0.05a
B	61.23a	24.41a	4.35a	4.31a	1.65a	1.01a	0.44a	0.13a	0.14a	0.04a
T	65.90a	20.32a	5.87a	4.35a	0.59b	0.43a	0.33a	0.08a	0.00a	0.01a
G	71.60a	14.50a	2.98a	5.53a	0.55b	0.24a	0.55a	0.00a	0.04a	0.03a
M	64.98a	21.13a	4.73a	4.67a	0.40b	0.35a	0.46a	0.13a	0.03a	0.00a

CK, J, B, T, G, and M represent no increase in drip irrigation amount, an increase in drip irrigation amount from the sowing stage to the jointing stage, from the jointing stage to the big trumpet stage, from the big trumpet stage to the tasseling stage, from the tasseling stage to the grain filling stage, and from the grain filling stage to the maturity stage of maize, respectively. In the same column, different small letters refer to differences among treatments at *p* < 0.05 level.

Overall, *ascomycetes*, *basidiomycetes*, *chytridomycetes*, and *mortieromycetes* together accounted for more than 94.00% of the total fungal abundance, indicating that these four groups were the dominant fungal communities in the soil under each treatment.

### 3.6 Correlation and redundancy analysis

**Figure 5:**
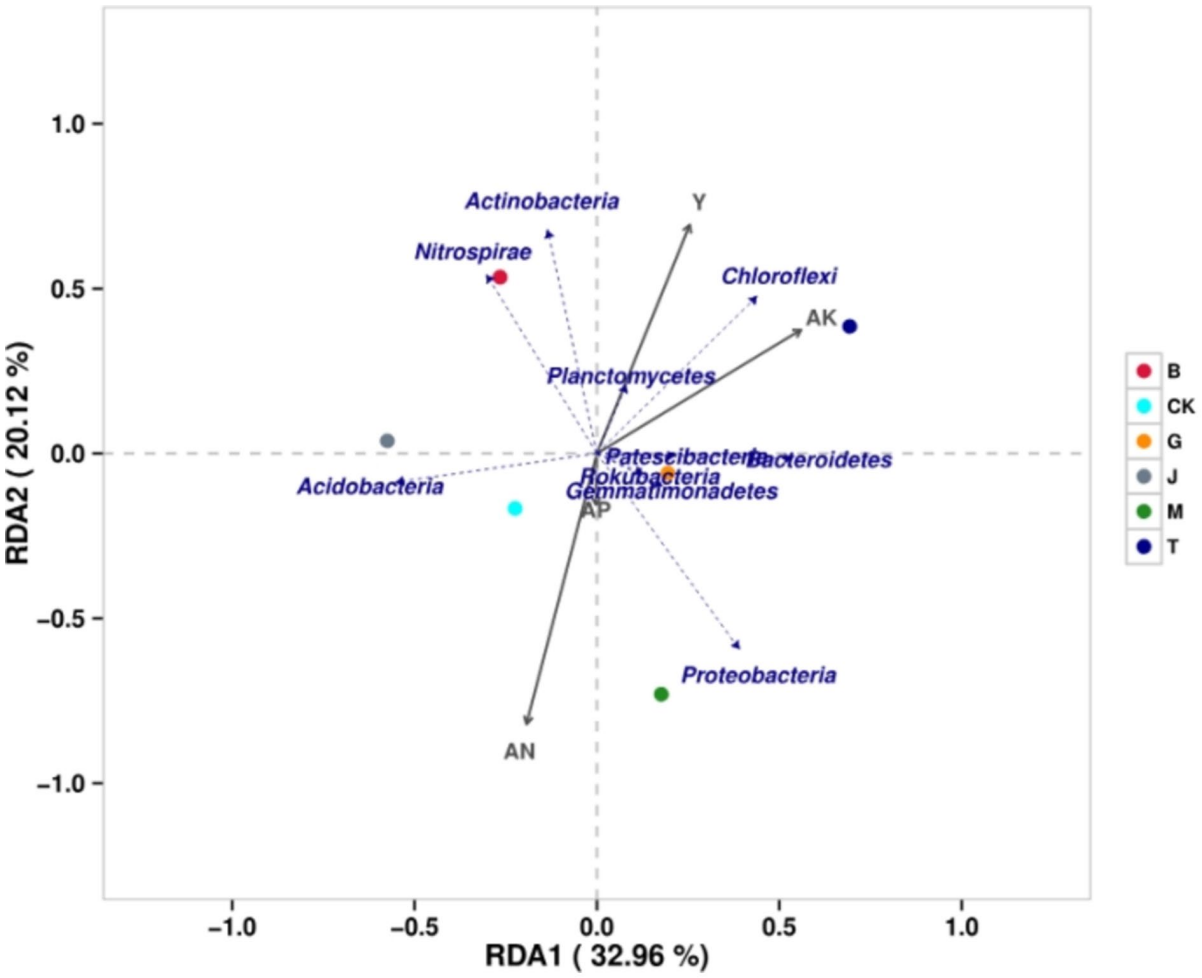
The redundancy analysis of bacterial community and related factors. CK, J, B, T, G, and M represent no increase in drip irrigation amount, an increase in drip irrigation amount from the sowing stage to the jointing stage, from the jointing stage to the big trumpet stage, from the big trumpet stage to the tasseling stage, from the tasseling stage to the grain filling stage, and from the grain filling stage to the maturity stage of maize, respectively.

The similarity ranking between each treatment and CK, from highest to lowest, was J, G, B, M, and T (Figure 5). The lowest similarity between each treatment was J and T. The similarity between the B and M treatments was only slightly higher than that between the J and T treatments. At the phylum level, the degree of influence, from greatest to least, was in the order of available nitrogen, maize grain yield, potassium, and phosphorus. The grain yield of maize was negatively correlated with *Proteobacteria* and *Acidobacteria* but positively correlated with other bacterial phyla. Available nitrogen was positively correlated with *Proteobacteria*, *Acidobacteria, Gemmatimonadetes,* and *Rokubacteria* but negatively correlated with other bacterial phyla. Available phosphorus was positively correlated with *Proteobacteria, Acidobacteria, Gemmatimonadetes, Bacteroidetes, Rokubacteria,* and *Patescibacteria* but negatively correlated with other bacteria. Available potassium was negatively correlated with *Acidobacteria* and *Nitrospirae* but positively correlated with other phyla. It could be seen that the four soil chemical properties were positively correlated with *Gemmatimonadetes* and *Rokubacteria*.

**Figure 6:**
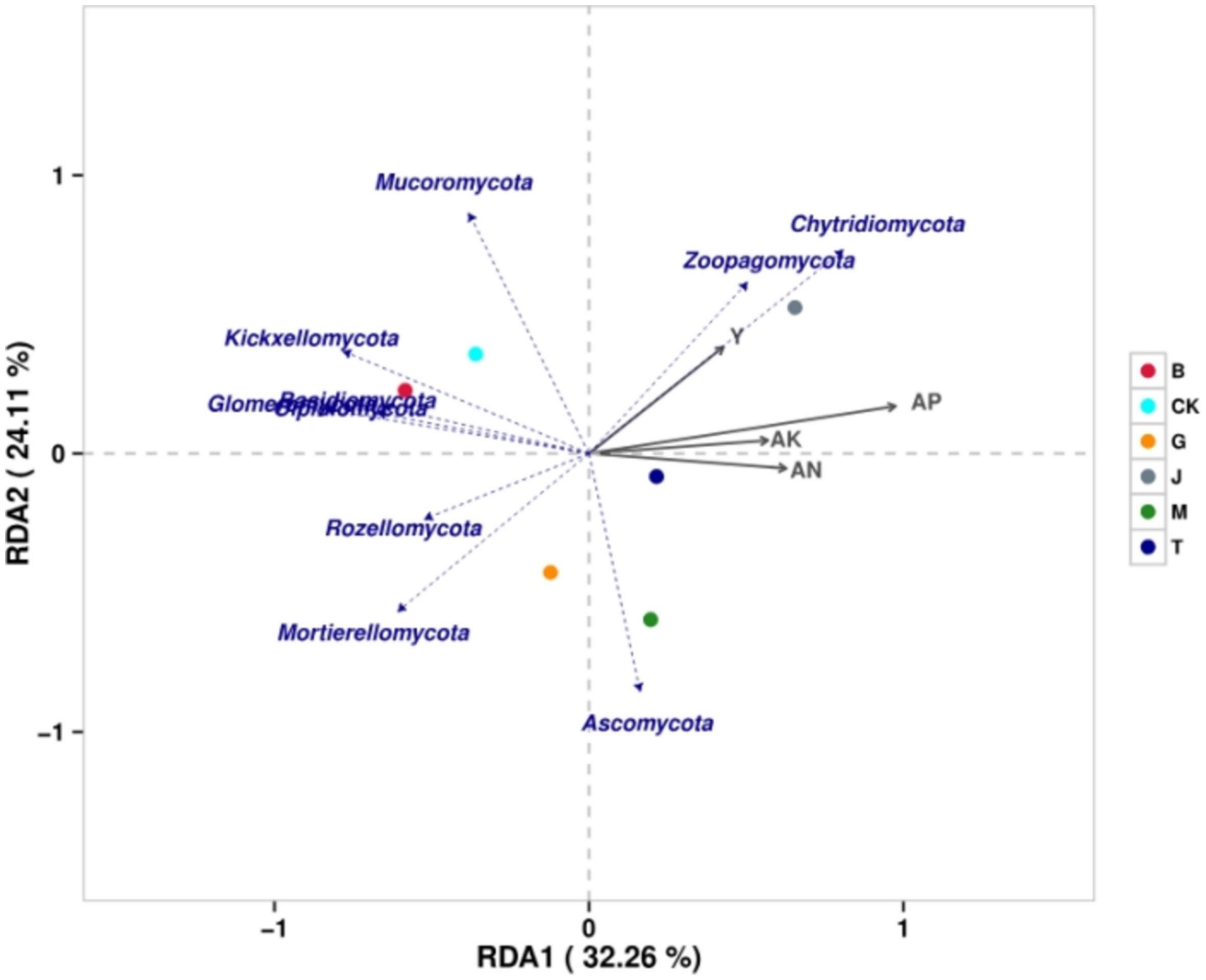
The redundancy analysis of the fungal community and related factors. CK, J, B, T, G, and M represent no increase in drip irrigation amount, an increase in drip irrigation amount from the sowing stage to the jointing stage, from the jointing stage to the big trumpet stage, from the big trumpet stage to the tasseling stage, from the tasseling stage to the grain filling stage, and from the grain filling stage to the maturity stage of maize, respectively.

The similarity between each treatment and CK was B, T, G, M, and J from the highest to the lowest (Figure 6). The similarity between the J and B treatments was the lowest. The similarity between the J and G treatments was slightly higher than that between the J and B treatments. The degree of influence on fungi was as follows: available phosphorus > available nitrogen > available potassium > maize grain yield. The grain yield was positively correlated with *Chytridiomycota*, *Zoopagomycota,* and *Mucoromycota* but negatively correlated with other phyla. Available nitrogen, available phosphorus, and available potassium were positively correlated with *Ascomycota*, *Chytridiomycota,* and *Zoopagomycota* but negatively correlated with other phyla. In conclusion, all four soil chemical properties were positively correlated with *Chytridiomycota* and *Zoopagomycota* but negatively correlated with *Basidiomycota, Mortierellomycota, Glomeromycota, Kickxellomycota, Rozellomycota,* and *Olpidiomycota.*

## 4 Discussion

Drip irrigation systems can accurately deliver available nutrients to the crop root zone, thereby increasing the efficiency of fertilizer use (Fan et al., 2017). In this study, the T treatment significantly increased soil-available potassium content compared to other treatments. This finding suggests that increasing irrigation from the maize tasseling to the grain-filling stage could enhance soil-available potassium content. The soil moisture status directly or indirectly influences the community structure and diversity of bacteria and fungi, which in turn affects the metabolic processes and survival strategies of microbial cells (Zhang et al., 2024).

*Chloromycetes* are autotrophic bacteria with diverse nutritional modes and metabolic pathways, contributing to the biogeochemical cycling of a series of important biogenic elements such as C, N, and S (Xian et al., 2019), whereas *Bacteroidetes* are moderately halophilic bacteria. In this study, the relative abundance of *chloromyces* and *Bacteroides* in the J treatment was the lowest. The change in the relative abundance of bacteria may be because of loss of aeration in the presence of excess water. Yuwen (2024) concluded that *chloromyces* abundance was negatively correlated with soil water content, while Bacteroides abundance was positively correlated with soil weight, which could explain this phenomenon.

*Actinomycetes* and *Nitrospira* both have certain biodegradation capabilities (Yu et al., 2020). In this study, available potassium was negatively correlated with *Acidobacteria* and *Nitrospirae* of soil, indicating that a reduction in *Acidobacteria* and *Nitrospirae* in the soil could lead to an increase in the amount of available potassium in the soil. *Actinomycetes* are one of the most widely distributed bacterial phyla in soil (Bao et al., 2021). As oligotrophic *bacteria*, *Actinomycetes* can form sporangium and exist in the environment and have a strong ability to degrade benzoic acid and p-hydroxybenzoic acid (Mao et al., 2010) and to sequester nitrogen (Hao et al., 2024). Moreover, Actinomycetes can stimulate crop growth and induce resistance to plant pathogens (Swarnalakshmi et al., 2016). *Pontomyces* are involved in the degradation of biopolymers from plant and fungal cell walls and have chitin-degrading capabilities (Kulichevskaya et al., 2019). In this study, the relative abundance of *blastomonas* in B treatment was the lowest. The relative abundance of actinomyces, *pontomyces,* and *nitrocellulosis* was the highest, indicating that increasing the drip irrigation amount from the stage of maize to the stage of large trumpet enhanced soil microbial and crop nitrogen fixation and disease resistance and reduced the content of absorbable vitamins.

*Rokubacteria* is a newly identified soil bacterium characterized by genes for synthesizing secondary metabolites, which may aid in iron resource competition and antibiotic production, demonstrating significant biosynthesis potential (Crits-Christoph et al., 2018). In this research, the augmentation of *Rokubacteria* in the soil can enhance maize yields and augment the quantity of available nitrogen, phosphorus, and potassium. Moreover, the relative abundances of *Rokubacteria* were the highest under M treatment, indicating that increasing drip irrigation from the filling to the maturity stage of maize increased soil fertility.

## 5 Conclusion

Increasing irrigation at any maize growth stage was conducive to a higher soil microbial OTU count. The number of fungal OTUs was significantly lower than that of bacterial OTUs, amounting to approximately one-fourth of the bacterial count.

Irrigation from the large trumpet stage to the filling stage of maize significantly increased soil bacterial richness.

Increasing irrigation during any maize growing period influenced the composition of soil fungal communities. Increased irrigation from the large trumpet stage to the heading stage of maize had a good effect on increasing soil-available nutrients.

## Data Availability

The raw data supporting the conclusions of this article will be made available by the authors, without undue reservation.
